# Physical restraint use in a United States intensive care unit—a retrospective cross sectional, single center cohort study from 2008 to 2022

**DOI:** 10.1016/j.lana.2026.101374

**Published:** 2026-01-14

**Authors:** Maximin Lange, Leo A. Celi, Ben Carter, Jesse D. Raffa, Sharon C. O'Donoghue, Marzyeh Ghassemi, Tom J. Pollard

**Affiliations:** a Massachusetts Institute of Technology, Cambridge, MA, USA; b Department of Psychosis Studies, Institute of Psychiatry, Psychology & Neuroscience, King's College London, London, UK; c Division of Pulmonary, Critical Care and Sleep Medicine, Beth Israel Deaconess Medical Center, Boston, MA, USA; d Department of Biostatistics, Harvard T H Chan School of Public Health, Boston, MA, USA; e Department of Biostatistics and Health Informatics, Institute of Psychiatry, Psychology & Neuroscience, King's College London, London, UK; f Beth Israel Deaconess Medical Center (Retired), Boston, MA, USA

**Keywords:** Physical restraints, Intensive care units, Health disparities, Racial equity, Critical care, Healthcare policy, Patient safety, Temporal trends, Electronic health records, Quality of care

## Abstract

**Background:**

Physical restraints are widely used in intensive care units (ICUs) despite uncertain clinical benefit and risks. We aimed to characterise patterns of restraint use, demographic and clinical predictors, and temporal trends before and after introduction of federal restraint-related reporting requirements.

**Methods:**

We conducted a retrospective cross-sectional study of 51,838 adults admitted to ICUs at Beth Israel Deaconess Medical Center, Boston, MA, USA, between 2008 and 2022, using data from the Medical Information Mart for Intensive Care IV (MIMIC-IV) electronic health record repository. Primary outcome was the proportion of ICU days with documented physical restraint use. Associations between restraint use and demographic and clinical factors were estimated using a binomial generalised linear model with a logit link. Propensity score matching compared Black and White patients under varying adjustment specifications.

**Findings:**

Among 51,838 patients (mean age 63.8 years; 57% male), 21,091 (40.7%) experienced restraint. Use increased from 36.9% in 2008–10 to 44.0% in 2020–22 (p < 0.0001). Asian (aOR 0.84, 95% CI 0.79–0.89) and Hispanic/Latino patients (aOR 0.87, 95% CI 0.83–0.92) had lower odds of restraint than White patients. Propensity score matching between Black and White patients revealed ethnic patterns were highly sensitive to model specification: excluding demographic characteristics revealed significant disparities, which were attenuated when psychiatric diagnoses were also excluded. Matched White patients were not representative of all White ICU patients but rather a subset resembling Black patients on observed characteristics.

**Interpretation:**

Restraint practices appear to vary with patient acuity, institutional factors, and communication barriers. The sensitivity of ethnic disparities to psychiatric diagnosis adjustment suggests these diagnoses may function as mediators rather than confounders, potentially reflecting systematic differences in clinical assessment along the causal pathway between ethnicity and restraint decisions. The non-representativeness of matched cohorts underscores that disparities depend on which patient subgroups are compared. Prospective multisite studies with standardized assessment protocols are needed to validate findings, disentangle true clinical variation from systematic bias and provide a more comprehensive understanding of restraint practices across US ICU settings.

**Funding:**

No study-specific funding was received.

**Research in context**

**Evidence before this study**

We searched PubMed, Web of Science, and Scopus from database inception to September 2025 using search terms including “physical restraint”, “mechanical restraint”, “intensive care”, “ICU”, “critical care”, “racial disparities”, “ethnic disparities”, and “health equity”, without language restrictions. We included observational studies, clinical trials, and systematic reviews examining physical restraint use in adult intensive care settings, with particular focus on studies reporting demographic disparities or temporal trends. Prior evidence primarily focused on emergency department settings, where Black patients tend to have higher odds of physical restraint use compared to other ethnic groups, while Hispanic patients were less likely to be restrained than non-Hispanic patients. The most recent examination of intensive care units (ICUs) restraint patterns in the United States only included non-intubated ICU patients and had limited sample size. A recent global meta-analysis found ICU restraint prevalence of approximately 40%, but provided minimal data on racial or ethnic disparities. In 2014, the Centers for Medicare and Medicaid Services (CMS) introduced Form CMS-10455 requiring hospitals to report deaths associated with restraint use, but we found no studies having evaluated the impact of this policy on restraint practices or disparities.

**Added value of this study**

This study provides the most comprehensive examination of ethnic disparities in ICU physical restraint use in the United States to date. We analyzed over a decade of electronic health record data to assess whether demographic disparities persist after extensive adjustment for clinical factors. We further examined temporal trends spanning the implementation of federal reporting requirements for restraint associated deaths, allowing us to assess whether this regulatory change was associated with improvements in practice patterns.

**Implications of all the available evidence**

Physical restraint use in intensive care units remains understudied despite evidence of it being widespread and a persistent challenge in the United States (US). Demographic, clinical, and organisational factors are associated with substantial variation in practice, with reliance on restraints differing by staffing levels, availability of de-escalation alternatives, institutional culture, and communication barriers. More research across US ICUs is needed to clarify causal pathways and guide equitable restraint-reduction efforts.

## Introduction

Physical restraints in healthcare settings serve as a safety measure for both patient and clinical staff.^1^^,^^2^ Risks from restraints are severe and potentially lethal.^3^, ^4^, ^5^, ^6^, ^7^

A number of professional and academic institutions have started to recommend a reduction of restraint use across all patient populations.^1^^,^^8^^,^^9^ In 2014, the Centers for Medicare & Medicaid Services (CMS) introduced Form CMS-10455,^10^ which required hospitals to report deaths associated with restraint and/or seclusion.

Recent investigations focused on understanding disparities in ethnicity, sex, gender and consequential risk of restraint.^11^, ^12^, ^13^, ^14^, ^15^ Most investigations on the topic have been conducted in an emergency department setting. A recent meta-analysis^16^ concluded that Black patients were at a higher risk of being physically restrained in relation to other ethnic groups, while Hispanic patients were less likely to be restrained compared with non-Hispanic patients.

Since Minnick,^17^ who examined ICU restraint patterns between 2003 and 2005, there has been little investigation into the prevalence and determinants of restraint use in United States ICUs. The only recent evidence comes from McGowan^18^ who studied racial and language differences in restraint use among non-intubated patients across five ICU units within the University of California, San Francisco hospital system from 2013 to 2022. They reported higher restraint odds for Black patients and for patients whose preferred language was neither English nor Spanish.

To extend this emerging evidence, particularly by examining a larger and more clinically diverse cohort over a longer period, and evaluating temporal and policy-related trends, we aimed to characterise patterns of restraint use, identify demographic and clinical predictors, and assess temporal trends before and after the introduction of federal restraint-related reporting requirements.

## Methods

A protocol with statistical analysis plan was uploaded^19^ Apr 23rd, 2025 prior to first accessing the data on June 20th 2025 to address this research question.

We conducted a retrospective cross-sectional study using electronic health records from Beth Israel Deaconess Medical Center, Boston, MA, USA. The study included all unique ICU patients aged ≥18 available on the MIMIC-IV^20^ v3.1 electronic health records database between 2008 and 2022, with any chief complaint. For participants with multiple ICU admissions during the study period, only the index admission was used.

Patient race and ethnicity were self reported. We use the following terminology: non Hispanic Black patients (hereafter, Black), non Hispanic White patients (hereafter, White), Hispanic or Latino patients of any race (hereafter, Hispanic/Latino), Asian patients (hereafter, Asian), and patients who declined to specify, selected “Other,” or had missing race/ethnicity data (hereafter, Unknown/Other). These categories align with standard epidemiological research practices while acknowledging the limitations of administratively collected race and ethnicity data.

For study inclusion, patients had to be 18 years of age or older and admitted to any ICU unit for a minimum duration of 24 h. Restraint days were identified from clinical notes and nursing documentation containing mentions of physical restraint, four-point restraint (including variations ‘4-point’), wrist restraint, soft restraint, leather restraint, or the ICD9 or ICD10 code for physical restraint utilization (V4987 & Z781, respectively); Otherwise, patients belonged to the ‘non-restraint’ group.

The primary outcome was the proportion of ICU days with physical restraint use at patient level calculated as:

*(Number of days restrained/Total ICU length of stay) × 100%*

A “restraint-documented day” was defined as any unique calendar date during the ICU stay on which at least one chart event matching the restraint keywords occurred. Secondary outcomes included binary restrain utilisation (yes/no) and binary death within 24 h of restraint (yes/no).

### Statistical analysis

#### Main analysis

For our primary model, we employed a binomial generalized linear model with a logit link function. We modelled the number of days with restraint as a binomial outcome with the number of trials equal to their total ICU length of stay.

The following covariates were used in the model building process. All were collected during day 1 of ICU admission, except for ICD codes, as diagnoses were assigned at discharge for billing purposes and encompass conditions identified throughout the entire hospitalization, not exclusively those present at ICU admission: Ethnicity; Age (continuous variable); Sex (male/female); Intubation status (yes/no); Lines on body (central/arterial/dialysis/external ventricular drain (EVD)/extracorporeal membrane oxygenation (ECMO)); Severity of Illness (Sequential Organ Failure Assessment (SOFA), Oxford Acute Severity of Illness Score (OASIS), Acute Physiology Score III (APSIII); Non-invasive ventilation mask (yes/no); English proficiency (yes/no); Level of agitation and sedation (Richmond Agitation–Sedation Scale, RASS/Confusion Assessment Method (CAM); Attempted suicide (yes/no); Chemical restraint received (haloperidol, droperidol, ziprasidone, olanzapine, ketamine, propofol, benzodiazepine, oxazepam, temazepam, clonazepam, alprazolam, midazolam, lorazepam, diazepam, dexmedetomidin, diphenhydramine); Time period (categorical three-year intervals: 2008–2010, 2011–2013, 2014–2016, 2017–2019, 2020–2022); ICU type (Cardiac Vascular, Coronary Care, Medical, Medical/Surgical, Neuro Intermediate, Neuro Stepdown, Neuro Surgical, Surgical, Trauma, Other); Path of admission (emergency, elective, transfer); Pain scale (regular, Critical-Care Pain Observation Tool (CPOT); Alcohol (Clinical Institute Withdrawal Assessment for Alcohol for Alcohol Withdrawal (CIWA/CIWA-AR); Presence of mental health diagnoses Psychosis (International Classification of Diseases (ICD) version 10, codes F2′, ICD Version 9 codes 295′, 297′, 298′), Mania/bipolar (ICD10 F30′, F31′, ICD9 296.0′, 296.1′, 296.4′, 296.5′, 296.6′, 296.7, 296.8′), Substance use (ICD10 F1′, ICD9 303′, 304′), Mental disorder due to physiological conditions (ICD10 F0′, ICD9 293′)).

Results are presented as adjusted odds ratios (aOR) with 95% confidence intervals (95%CI) and p-values, with statistical significance set at 0.05.

Starting from a minimal model (including age, gender, ethnicity, ICU type, invasive ventilation status, deep sedation status, chemical restraint use, delirium assessment, we added clinically meaningful variables one at a time based on expert consultation (Authors LAC and SCOD). Variables were retained if they met statistical significance (p < 0.05) and improved model fit as measured by Akaike's Information Criterion (AIC) and the Hosmer–Lemeshow goodness-of-fit test. We then removed variables that were not statistically significant and did not materially improve model fit.

To examine temporal trends in restraint use, we additionally calculated the proportion of ICU days with restraint use for each three-year interval across the study period. The Cochran–Armitage test for trend was used to assess the statistical significance of changes in proportions over time.

We also assessed two-way interaction terms between ethnicity and key variables (mental health diagnoses, intubation status, and time period). Interaction terms were added individually and retained based on AIC comparisons to evaluate model improvement.

### Secondary and sensitivity analyses

We conducted two secondary analyses using logistic regression with the same set of predictor variables as in the final main model. The first examined binary restraint utilization (any documented restraint during the ICU stay: yes/no). The second examined death within 24 h of a restraint event (yes/no), restricted to patients who had experienced any restraint.

As a sensitivity analysis, we stratified the main analysis by ICU type to account for variation in restraint practices across different unit types. We also performed 1:1 propensity score matching comparing Black/African American and White patients, treating race as the exposure. This comparison was pre-specified in the study protocol because these were the two largest racial groups in the cohort, providing the most stable matched samples. The final binomial generalized linear model was then re-estimated in the matched cohort, using the same variables as in the primary analysis. To assess whether these factors functioned as confounders or mediators, two additional specifications were examined: (i) excluding demographics (age, sex, preferred language), and (ii) excluding both demographics and psychiatric or behavioural diagnoses. Within each matched cohort, we re-fit the primary binomial model and retained a binary Black-vs-White indicator to estimate the adjusted odds ratio for restraint exposure under each specification. Lastly, we investigated alternative definitions of restraint use based on varying documentation patterns to evaluate the robustness of our findings. The primary analysis used a broad text-search definition of physical restraint designed to maximise sensitivity. Any documentation within the ICU stay was classified as a restraint event if the chart text contained terms related either to restraint application (e.g., “restrain,” “restrained,” “restraint(s),” “wrist restraint,” “soft restraint,” “leather restraint”) or to potentially ambiguous descriptors frequently associated with restraint use (e.g., “tied,” “secured,” “immobilized,” “extremities secured,” “limbs secured,” “4-point,” “four-point”). In sensitivity analyses, we applied a more conservative definition restricted to unambiguous restraint terminology. This stricter definition required explicit phrases such as “restraint,” “restrained,” “restraints,” “wrist restraints,” “soft restraints,” “limb restraints,” “bilateral wrist restraints,” “4-point restraints,” or “non-violent/NV restraints,” and excluded general securement language (e.g., “secured,” “tied,” “immobilized”) and non-specific uses of “4-point” without reference to restraint. By removing terms that may reflect device or line securement rather than physical restraint, this conservative definition isolates clearly documented restraint episodes.

### Role of the funding source

No specific funding was obtained for the delivery of this study. The funding sources supporting individual authors had no role in study design; data collection, analysis, or interpretation; manuscript writing; or the decision to submit this manuscript for publication. Authors were not precluded from accessing data in the study, and they accept responsibility to submit for publication.

## Results

We screened 364,627 unique patients for study eligibility. 51,838 patients fulfilled our inclusion criteria, of which 21,091 (40.7%) involved at least one physical restraint episode and 30,747 (59.3%) did not (Table 1). Patients were older adults (mean age 63.8 years, SD 16.6), with longer stays more common among restrained patients. The cohort was predominantly male (57%, of which 42% were restrained). 66% were White (39% of these restrained), 9% Unknown/Other (49% restrained), 9% Black (39% restrained), 3% Hispanic/Latino (38% restrained), and 3% Asian (37% restrained). Restraint use was rising over time, from 36.9% in 2008–10 to 44.0% in 2020–22.

**Table 1:** Patient profile.

Characteristic	Overall	Not restrained	Restrained
N	51,838	30,747	21,091
Age, mean (SD)	63.8 (16.6)	63.8 (16.4)	63.7 (17.0)
Age group			
18–30	2509	1416 (56.4%)	1093 (43.6%)
31–40	2784	1599 (57.4%)	1185 (42.6%)
41–60	14,519	8650 (59.6%)	5869 (40.4%)
61–75	18,193	11,099 (61.0%)	7094 (39.0%)
76 or older	13,833	7983 (57.7%)	5850 (42.3%)
Sex			
F	22,303	13,644 (61.2%)	8659 (38.8%)
M	29,535	17,103 (57.9%)	12,432 (42.1%)
Length of ICU stay (days)			
1–3 days	27,932	20,430 (73.1%)	7502 (26.9%)
4–6 days	14,165	7892 (55.7%)	6273 (44.3%)
7–10 days	5040	1708 (33.9%)	3332 (66.1%)
11–50 days	4618	709 (15.4%)	3909 (84.6%)
>50 days	83	8 (9.6%)	75 (90.4%)
Ethnicity			
Asian	1543	976 (63.3%)	567 (36.7%)
Black	4568	2767 (60.6%)	1801 (39.4%)
Hispanic/Latino	1970	1229 (62.4%)	741 (37.6%)
Unknown/Declined/Other	9760	4976 (51.0%)	4784 (49.0%)
White	33,997	20,799 (61.2%)	13,198 (38.8%)
Time period			
2008–2010	13,627	8597 (63.1%)	5030 (36.9%)
2011–2013	10,575	6193 (58.6%)	4382 (41.4%)
2014–2016	10,594	6094 (57.5%)	4500 (42.5%)
2017–2019	9874	5847 (59.2%)	4027 (40.8%)
2020–2022	7168	4016 (56.0%)	3152 (44.0%)

### Primary statistical model

During backward model refinement, sequentially removed for lack of statistical significance and minimal contribution to model fit were: suicide attempt diagnosis (p = 0.50), Black/African American race (p = 0.33), time period 2011–2013 (p = 0.29), and presence of dialysis line (p = 0.23). Invasive ventilation was associated with approximately double the odds of a higher restraint-day proportion (Table 2, aOR 2.03, 95% CI 1.95–2.12, p < 0.0001), while deep sedation was associated with 60% higher odds (aOR 1.60, 1.56–1.64, p < 0.0001). Patients receiving chemical restraint had more than twice the odds of greater restraint use (aOR 2.14, 2.08–2.20, p < 0.0001), and those with a positive CAM screen had similarly elevated odds (aOR 2.56, 2.50–2.62, p < 0.0001). The presence of an EVD or ECMO modestly raised odds by 17% (aOR 1.17, 1.13–1.21, p < 0.0001), and a central line by 29% (aOR 1.29, 1.23–1.35, p < 0.0001). Male sex was associated with 18% higher odds relative to females (aOR 1.18, 1.16–1.20, p < 0.0001). Non-English speakers similarly had higher odds (aOR 1.21, 1.18–1.26, p < 0.0001). Among mental-health–related factors, psychosis (aOR 1.24, 1.16–1.32), mania/bipolar disorder (aOR 1.10, 1.04–1.16), substance-use disorder (aOR 1.64, 1.60–1.68), and physical-condition–related mental disorder (aOR 1.94, 1.89–1.99) were each independently associated with greater restraint use (all p < 0.0001, except mania p = 0.0009). Increasing illness severity also predicted restraint: each one-point rise in SOFA (aOR 1.03, 1.02–1.03) or OASIS (aOR 1.03, 1.02–1.03) increased odds, whereas higher APS III scores were associated with slightly lower odds (aOR 0.99, 0.99–0.99, p < 0.0001). Older age was inversely related to restraint use (aOR 0.92, 0.91–0.93, p < 0.0001). Compared with White patients, those identifying as Asian (aOR 0.84, 0.79–0.89) and Hispanic/Latino (aOR 0.87, 0.83–0.92) had lower odds of restraint, whereas those of Unknown/Declined/Other ethnicity had modestly higher odds (aOR 1.07, 1.04–1.09; all p < 0.0001). Relative to the Cardiac Vascular ICU reference group, all other ICU types demonstrated greater restraint use, most prominently the Neuro Surgical ICU (aOR 7.81, 7.32–8.33), Neuro Intermediate/Step-down units (Neuro Intermediate aOR 4.93, 4.72–5.15; Neuro Step-down aOR 5.07, 4.71–5.45), and Medical/Surgical ICUs (Medical/Surgical aOR 3.66, 3.52–3.81; Medical aOR 3.78, 3.65–3.92; all p < 0.0001). Temporal trends showed a steady rise over time: 2014–2016 (aOR 1.44, 1.41–1.48), 2017–2019 (aOR 1.40, 1.36–1.44), and 2020–2022 (aOR 1.58, 1.53–1.63; all p < 0.0001).

**Table 2:** Multivariable analysis of physical restraint use–primary model.

Variable	aOR (95% CI)	p-value
**Patient characteristics**		
Male sex	1.18 (1.16–1.20)	<0.0001
Age	0.92 (0.91–0.93)	<0.0001
Non-English language	1.21 (1.18–1.26)	<0.0001
**Ethnicity (vs White)**		
Asian	0.84 (0.79–0.89)	<0.0001
Hispanic/Latino	0.87 (0.83–0.92)	<0.0001
Unknown/Declined/Other	1.07 (1.04–1.09)	<0.0001
**Clinical severity (per point)**		
SOFA score	1.03 (1.02–1.03)	<0.0001
OASIS score	1.03 (1.02–1.03)	<0.0001
APS III score	0.99 (0.99–0.99)	<0.0001
**Respiratory support & sedation**		
Invasive ventilation	2.03 (1.95–2.12)	<0.0001
Deep sedation	1.60 (1.56–1.64)	<0.0001
**Chemical restraint & delirium**		
Chemical restraint	2.14 (2.08–2.20)	<0.0001
Positive CAM screen	2.56 (2.50–2.62)	<0.0001
**Invasive procedures**		
EVD or ECMO	1.17 (1.13–1.21)	<0.0001
Arterial line	1.04 (1.01–1.07)	0.0031
Central line	1.29 (1.23–1.35)	<0.0001
**Mental health diagnoses**		
Psychosis	1.24 (1.16–1.32)	<0.0001
Mania/Bipolar	1.10 (1.04–1.16)	0.0009
Substance use	1.64 (1.60–1.68)	<0.0001
Physical-condition–related mental disorder	1.94 (1.89–1.99)	<0.0001
**ICU type (vs CVICU)**		
Coronary care unit	2.37 (2.27–2.47)	<0.0001
Medical ICU	3.78 (3.65–3.92)	<0.0001
Medical/Surgical ICU	3.66 (3.52–3.81)	<0.0001
Neuro intermediate	4.93 (4.72–5.15)	<0.0001
Neuro step-down	5.07 (4.71–5.45)	<0.0001
Neuro surgical ICU	7.81 (7.32–8.33)	<0.0001
Surgical ICU	4.27 (4.12–4.43)	<0.0001
Trauma SICU	3.12 (3.00–3.24)	<0.0001
Other ICU	5.92 (5.39–6.51)	<0.0001
**Time period (vs 2008**–**2010)**		
2014–2016	1.44 (1.41–1.48)	<0.0001
2017–2019	1.40 (1.36–1.44)	<0.0001
2020–2022	1.58 (1.53–1.63)	<0.0001

Model performance: *N = 51,838; AIC = 189,520; log-likelihood = –94,727; McFadden's Pseudo-R*^*2*^*=0.236; Deviance = 160,845; model converged successfully.*

### Temporal trend analysis

Over the 14-year observation period, the proportion of ICU days with recorded physical restraint increased from 27.9% in 2008–2010 to 48.0% in 2020–2022, indicating a consistent upward trajectory (Table 3). Cochran–Armitage test confirmed a statistically significant increasing trend in restraint use over time (Z = 79.0, p < 0.0001).

**Table 3:** Temporal trend analysis.

Time period	Total restraint days	Total ICU days	Proportion of ICU days restrained (%)
2008–2010	17,387	62,269	27.9
2011–2013	14,484	46,968	30.8
2014–2016	21,026	49,128	42.8
2017–2019	21,992	49,437	44.5
2020–2022	19,867	41,365	48.0

### Interaction effects

We found significant interactions between ethnicity and several clinical factors, indicating that the association between ethnicity and restraint use varied depending on patients' clinical characteristics (Table 4). Among psychosis patients, the association between being Asian and restraint use was 3.74 times stronger than among White patients (interaction aOR 3.74, 95% CI 1.90–7.35, p = 0.0001). Conversely, among patients with bipolar or mania diagnoses, the association between being Asian and restraint use was 78% weaker than among White patients (aOR 0.21, 0.07–0.66, p = 0.0076). For patients with a mental disorder due to a physical condition, where the association between being Asian and restraint use was attenuated (aOR 0.66, 0.55–0.80, p < 0.0001). Among patients receiving invasive ventilation, the association between being Hispanic/Latino and restraint use was 30% stronger than among White patients (aOR 1.30, 1.03–1.63, p = 0.023). However, among those with bipolar/mania (aOR 0.56, 0.38–0.82, p = 0.0034) or substance-use disorder (aOR 0.84, 0.73–0.95, p = 0.0091), the association between being Hispanic/Latino and restraint use was significantly weaker compared with White patients. For patients of Unknown, Declined, or Other ethnicity, similar patterns emerged, where the association between being of these ethnicities and restraint use was 28% weaker among those with bipolar/mania (aOR 0.71, 0.63–0.81, p < 0.0001) and 23% weaker among those with a mental disorder due to a physical condition (aOR 0.77, 0.72–0.82, p < 0.0001), whereas the association was slightly stronger among those with substance-use disorder (aOR 1.08, 1.02–1.14, p = 0.0078). Temporal interactions were also evident: among patients of Unknown, Declined, or Other ethnicity from 2017 to 2019 and 2020–2022, the association between ethnicity and restraint use was 18% and 13% stronger, respectively, than the corresponding temporal trend among White patients (aOR 1.17, 1.10–1.25, p < 0.0001; aOR 1.12, 1.05–1.19, p = 0.0002).

**Table 4:** Interaction effects.

Interaction	aOR (95% CI)	p-value
Asian × Psychosis	3.74 (1.90–7.35)	0.0001
Asian × Bipolar/Mania	0.21 (0.07–0.66)	0.0076
Asian × Physical-condition mental disorder	0.66 (0.55–0.80)	<0.0001
Hispanic/Latino × Bipolar/Mania	0.56 (0.38–0.82)	0.0034
Hispanic/Latino × Substance use	0.84 (0.73–0.95)	0.0091
Hispanic/Latino × Ventilation	1.30 (1.03–1.63)	0.023
Unknown/Other × Bipolar/Mania	0.71 (0.63–0.81)	<0.0001
Unknown/Other × Substance use	1.08 (1.02–1.14)	0.0078
Unknown/Other × Physical-condition mental disorder	0.77 (0.72–0.82)	<0.0001
Unknown/Other × 2017–2019	1.17 (1.10–1.25)	<0.0001
Unknown/Other × 2020–2022	1.12 (1.05–1.19)	0.0002

### Secondary outcome analyses

#### Binary restraint use (yes/no)

After adjustment for ventilation, sedation, chemical restraint, delirium, invasive lines, gender, mental health diagnoses, language, severity scores, ICU type, and time period, demographic effects were modest. Hispanic/Latino patients had slightly lower odds of restraint use than White patients (Supplementary Table S3; aOR 0.89, 95% CI 0.79–0.99, p = 0.045), while Asian patients showed no significant difference (aOR 0.94, 95% CI 0.83–1.07, p = 0.37). Patients of Unknown, Declined, or Other ethnicity had higher odds (aOR 1.15, 95% CI 1.09–1.22, p < 0.0001). The strongest clinical correlates were delirium (aOR 2.71, 95% CI 2.55–2.87), a mental disorder secondary to a physical condition (aOR 2.48, 95% CI 2.30–2.67), chemical restraint use (aOR 1.94, 95% CI 1.84–2.05), and deep sedation (aOR 1.79, 95% CI 1.69–1.89). Neurological ICUs again showed the highest odds of restraint, Neuro Surgical ICU (aOR 4.47, 95% CI 3.84–5.19) and Neuro Stepdown units (aOR 4.01, 95% CI 3.40–4.74), indicating substantial practice variation across settings. Adjusted temporal effects demonstrated a gradual decline after 2014, with 24% lower odds in 2017–2019 (aOR 0.76, 95% CI 0.72–0.81) and 12% lower odds in 2020–2022 (aOR 0.88, 95% CI 0.83–0.95) compared with 2008–2010.

#### Death within 24 h of restraint (yes/no)

Death within 24 h of restraint occurred in 242 cases out of all 21,091 restrained patients, corresponding to an incidence of 1.15%. The logistic model restricted to restrained patients showed high discrimination (Receiver Operating Characteristic—Area Under the Curve = 0.85) and good calibration (Brier = 0.011). After adjustment for ventilation, sedation, chemical restraint, delirium, invasive lines, gender, mental health diagnoses, language, severity scores, ICU type, and time period, no significant ethnic or temporal effects were observed, indicating that restraint related mortality risk was primarily driven by physiological rather than demographic factors. Increasing age (Supplementary Table S4; aOR 1.65, 95% CI 1.39–1.96, p < 0.0001), higher acute physiology scores (APS III aOR 1.02 per point, p < 0.0001; SOFA aOR 1.07 per point, p = 0.0030), and deep sedation (aOR 1.38, 95% CI 1.00–1.90, p = 0.047) were associated with higher odds of death. In contrast, delirium (aOR 0.61, 95% CI 0.44–0.84, p = 0.0031), chemical restraint (aOR 0.64, 95% CI 0.42–0.97, p = 0.039), and mental disorders secondary to physical illness (aOR 0.32, 95% CI 0.18–0.57, p < 0.0001) were associated with lower odds. Mortality risk was substantially elevated in neurological and medical ICUs, particularly the Neuro Surgical ICU (aOR 18.64, 95% CI 7.17–48.46).

### Sensitivity analyses

To examine unit-level heterogeneity in restraint practices, the final binomial model was stratified by ICU type while retaining the same demographic and clinical predictors. Core clinical associations were directionally consistent across all units: positive delirium screening, deep sedation, chemical restraint, and mechanical ventilation remained the strongest drivers of restraint use. However, the strength of these associations varied substantially by ICU (Supplementary Table S5). Model fit was highest in the neuro-intermediate (pseudo-R^2^ = 0.378) and neuro-stepdown (pseudo-R^2^ = 0.365) units, indicating that covariates explained a large proportion of restraint variation in these settings, and lowest in the cardiac vascular ICU (pseudo-R^2^ = 0.077) and medical–surgical ICU (pseudo-R^2^ = 0.243). Severity indicators (SOFA, OASIS, APS III) showed consistent positive associations across strata, indicating that patients with higher acute illness severity were more likely to be restrained. Mental disorders secondary to physical illness and substance-use disorders were strongly associated with restraint across all ICUs, whereas psychosis and bipolar/mania diagnoses were less consistent. Demographic effects were modest but heterogeneous: male gender and non-English language preference increased restraint odds in most units, while Asian and Hispanic/Latino ethnicity tended to be associated with lower odds in cardiac and medical ICUs but occasionally reversed in neuro-surgical units. Temporal terms showed declining restraint use in cardiac and coronary ICUs after 2014 but rising odds in surgical and trauma ICUs. Overall, the same clinical determinants underpinned restraint use across units, though their magnitude and demographic gradients differed markedly by ICU environment.

After 1:1 propensity score matching of 4025 Black/African American and 4025 White ICU patients, we re-estimated the full multivariable model including race as an explicit covariate. Matched White patients differed substantially from the broader unmatched White population across multiple characteristics (Supplementary Table S6), being younger, less often admitted from elective sources, and more frequently admitted to higher-acuity ICUs such as medical or neuro-intermediate units. Black race was not associated with higher odds of physical restraint (Supplementary Table S7, aOR 1.00, 95% CI 0.96–1.05, p = 0.68), indicating no residual racial effect after balancing on demographic, clinical, and contextual factors. When demographics (age, sex, preferred language) were excluded from propensity score matching but retained in the outcome model, Black race became significantly associated with restraint use (aOR 1.14, 95% CI 1.08–1.19, p < 0.0001), suggesting that demographic imbalances between racial groups contribute to observed disparities. However, when both demographics and psychiatric or behavioural diagnoses were excluded from propensity score matching (but retained in the outcome model), the race effect returned to non-significance (aOR 1.01, 95% CI 0.96–1.06, p = 0.63). When applying alternative restraint documentation with a stricter set of terms to isolate definite restraint events (e.g., specific phrases including the word “restraint”) analysis overall restraint prevalence decreased from 40.69% to 40.60%. 43 patients (0.08%) were reclassified from restrained to not restrained.

Our protocol included a planned complete case analysis; however, this was not required since no patients had missing data on any predictors or outcomes.

## Discussion

We present a comprehensive analysis of physical restraint disparities in intensive care units in a major US medical center, examining over 51,000 patients across a 14-year period. Our findings reveal ethnic disparities in restraint use and increasing restraint utilization over time.

We present a 40.7% overall restraint rate, substantially higher than emergency department studies but almost an exact overlap with a recent meta-analysis of restraint rates in ICUs globally.^21^ We found known factors associated with restraint, including mechanical ventilation and delirium.^17^^,^^22^, ^23^, ^24^, ^25^ Our report of language preference to be independently associated with restraint use is in concordance with recent findings from other institutions in the US.^18^

Most importantly, we identified lower odds for Asian and Hispanic/Latino patients compared to White patients, even after extensive adjustment for clinical severity, mental health diagnoses, and ICU practices. However, these lower odds were substantially modified by clinical context, with certain combinations of ethnicity and psychiatric diagnosis or respiratory support status showing markedly different patterns, indicating that demographic disparities in restraint use are complex and highly context dependent. Although the Black race variable was not retained in the final main-effects model following backward elimination, forcing this term back into the propensity-score–matched models revealed that Black race was not associated with higher odds of physical restraint after balancing on demographic, clinical, and contextual factors. However, when demographics were excluded from propensity-score matching, a significant racial disparity emerged. When both demographics and psychiatric or behavioural diagnoses were excluded from propensity matching, the racial effect again became non-significant. It is important to note that the matched White cohort was not representative of all White ICU patients but rather of a subset selected to closely resemble Black patients on observed characteristics.

Implementation of Form CMS 10455 was associated with a statistically significant increase in restraint use following policy implementation, with overall rates rising. Although the 2014 federal update primarily clarified reporting of deaths temporally associated with restraints/seclusion, these changes may have coincided with system-wide compliance efforts (e.g. staff re-training, EHR prompts/templates, audits, and risk-management oversight) that could have increased documentation of restraint use.

The lower odds we observed for Asian and Hispanic/Latino patients have not been consistently reported in previous ICU literature, though this may reflect the limited focus on racial disparities in critical care research. Lower odds for hispanic and latino patients have been reported in the emergency department.^12^ Our interaction analyses suggest these disparities are not uniform but vary significantly based on clinical context, with particularly pronounced effects in certain ICU types and among patients with specific diagnoses. Our findings suggest that reporting requirements alone may not be associated with changes in clinical practice. Rather than focusing solely on adverse event reporting, future policies could consider addressing the underlying factors associated with restraint use, including staffing ratios, alternative intervention availability, and institutional culture.^26^ Healthcare organizations should implement structured assessment protocols that require explicit justification for restraint use and regular reassessment of continued need. Each ICU may need to tailor these protocols to effectively address the specific needs of their patient population.^27^

Study strengths include a large sample size, comprehensive temporal analysis, and robust sensitivity analysis. Key limitations include single-center design, reliance on clinical documentation for restraint identification, and inability to assess the appropriateness of individual restraint decisions or access to alternative interventions. Another major limitation is our reliance on discharge ICD codes. These are assigned retrospectively and may include diagnoses made after restraint application, introducing reverse causality bias. Future prospective studies should capture psychiatric diagnoses present at ICU admission to establish true baseline risk factors. Future research should further examine restraint practices across multiple institutions to assess the generalizability of our findings. Lastly, electronic health record documentation is inherently subject to potential underreporting, misclassification, and variation in narrative detail, which could introduce measurement error.

## Contributors

LAC conceived the idea of investigating intensive care physical restraint prevalence. BC, JDR and ML developed the statistical analysis plan which was checked by TJP and LAC. ML conducted all statistical analysis, which were checked by BC, JDR and MG. ML designed all tables. ML wrote the first draft and all consecutive drafts of the manuscript. LAC and SCOD provided clinical guidance. TJP, BC, JDR, SCOD, MG and LAC read and commented on the manuscript. ML had final responsibility for the decision to submit the study for publication.

## Data sharing statement

The data used in this analysis are from the MIMIC-IV v3.1 database, which is hosted on PhysioNet and made available to qualified researchers upon completion of the required data use training and approval of a data use agreement (https://physionet.org). All variables used in this study, along with their definitions, are included in the publicly accessible MIMIC-IV data dictionary. SQL extraction code, data processing scripts and statistical analysis code copy are publicly available at: https://github.com/maximinl/restraints-mimic.

These materials will be permanently archived and openly accessible without restriction. To access the underlying EHR data, researchers must apply through PhysioNet, complete the appropriate training, and agree to the MIMIC-IV data use agreement. No additional restrictions apply beyond those required by PhysioNet.

A copy of the protocol with statistical analysis plan is publicly available.^19^

## Ethical approval

This research was conducted in accordance with the Declaration of Helsinki and followed international ethical standards for medical research involving human data. MIMIC-IV data was collected as part of routine clinical care. It has been deidentified and transformed. It is available to researchers who have completed training in data handling for human research and signed a data use agreement. It was approved for research by the institutional review boards of the Massachusetts Institute of Technology and Beth Israel Deaconess Medical Center, who granted a waiver of informed consent and approved the sharing of the research resource. Individual patient consent will not be required for publication as this was addressed in the original approval for the MIMIC-IV databases, which permits research use and publication with proper de-identification maintained.

## Declaration of interests

ML is supported by the London Interdisciplinary Social Science Doctoral Training Partnership (LISS DTP) and the Institution for Engineering and Technology (IET) and has received consulting fees from The London Psychiatry Clinic Limited, unrelated to this manuscript. BC is supported through the NIHR Maudsley Biomedical Research Centre at the South London and Maudsley NHS Foundation Trust in partnership with King's College London. The views expressed are those of the authors and not necessarily those of the NIHR or the Department of Health and Social Care. LAC is funded by the National Institute of Health through DS-I Africa U54 TW012043-01 and Bridge2AI OT2OD032701, the National Science Foundation through ITEST #2148451, and a grant of the Korea Health Technology R&D Project through the Korea Health Industry Development Institute (KHIDI), funded by the Ministry of Health & Welfare, Republic of Korea (grant number: RS-2024-00403047). JDR is supported by the NIH and Phillips Healthcare. MG reports institutional grants from the National Science Foundation, Robert Wood Johnson Foundation, Moore Foundation, Takeda, Volkswagen Foundation, Janssen, and Google, and unpaid leadership and fiduciary roles with AHLI and CHAI, all unrelated to this manuscript. TJP is supported through NIH grants R01EB030362 and OT2OD032701 as well as Korea Health Industry Development Institute (KHIDI), funded by the Ministry of Health & Welfare, Republic of Korea (grant number: RS-2024-00403047 and RS-2024-00439677).
