# Supplementary material for: Physical restraint use in a United States intensive care unit—a retrospective cross sectional, single center cohort study from 2008 to 2022
Source: Lancet Reg Health Am. 2026 Jan 14;55:101374. doi: 10.1016/j.lana.2026.101374 (PMC12829159; doi:10.1016/j.lana.2026.101374)
Supplement: Supplementary Tables [file mmc1.docx]

# **SUPPLEMENTARY MATERIAL FOR:**

# Physical Restraint Use in a United States Intensive Care Unit – A Retrospective cross sectional, single center cohort study using electronic health records from 2008 to 2022

Maximin Lange^1,2^ (ORCID 0000-0002-6253-8741), Leo A Celi^1,3,4^ (ORCID 0000-0001-6712-6626), Ben Carter^5^ (ORCID 0000-0003-0318-8865),

Jesse D Raffa^1^ (ORCID 0000-0001-8665-2429), Sharon C. O'Donoghue^6^ (ORCID 0009-0002-2136-026X) & Tom J Pollard^1^ (ORCID 0000-0002-5676-7898)

^1^ Massachusetts Institute of Technology, Cambridge, MA, USA

^2^ Department of Psychosis Studies, Institute of Psychiatry, Psychology & Neuroscience, King’s College London, London, UK

^3^ Division of Pulmonary, Critical Care and Sleep Medicine, Beth Israel Deaconess Medical Center, Boston, MA, USA

^4^ Department of Biostatistics, Harvard T H Chan School of Public Health, Boston, MA, USA

^5^ Department of Biostatistics and Health Informatics, Institute of Psychiatry, Psychology & Neuroscience, King’s College London, London, UK

^6^ Beth Israel Deaconess Medical Center (Retired), Boston, MA, USA

​​Corresponding author Maximin Lange

Email mlange2@mit.edu

Figures 0

Tables 6

**Software**

All analyses were conducted using Python in Google Colab. Primary statistical models (binomial GLM with logit link) were fitted using statsmodels (version 0.14.4) with maximum likelihood estimation via iteratively reweighted least squares (IRLS). Propensity score estimation used scikit-learn's LogisticRegression (version 1.6.1, parameters: C=1.0, max_iter=1000, random_state=42). Data manipulation used pandas (version 2.2.2), numerical computations used numpy (version 2.0.2), and visualization used matplotlib (version 3.10.0).

**Sample Size Considerations**

No a priori power calculations were performed as this is a retrospective analysis using all available data from the MIMIC-IV database the sample size was expected to be sufficient to detect clinically meaningful differences in restraint use across demographic groups.

**Suppl. Table 1:** Extended Table 1 from Main Manuscript

| **Characteristic** | **Overall** | **Not Restrained** | **Restrained** |
| --- | --- | --- | --- |
| **N** | **51838** | **30747** | **21091** |
|  |  |  |  |
|  |  |  |  |
| **Age, mean (SD)** | **63.8 (16.6)** | **63.8 (16.4)** | **63.7 (17.0)** |
|  |  |  |  |
| **Age Group** |  |  |  |
| **18–30** | **2509** | **1416 (56.4%)** | **1093 (43.6%)** |
| **31–40** | **2784** | **1599 (57.4%)** | **1185 (42.6%)** |
| **41–60** | **14519** | **8650 (59.6%)** | **5869 (40.4%)** |
| **61–75** | **18193** | **11099 (61.0%)** | **7094 (39.0%)** |
| **76** | **13833** | **7983 (57.7%)** | **5850 (42.3%)** |
|  |  |  |  |
| **Gender** |  |  |  |
| **F** | **22303** | **13644 (61.2%)** | **8659 (38.8%)** |
| **M** | **29535** | **17103 (57.9%)** | **12432 (42.1%)** |
|  |  |  |  |
| **Length of Stay (days)** |  |  |  |
| **1-3 days** | **31142** | **22535 (72.4%)** | **8607 (27.6%)** |
| **4-6 days** | **11699** | **6108 (52.2%)** | **5591 (47.8%)** |
| **7-10 days** | **4597** | **1457 (31.7%)** | **3140 (68.3%)** |
| **11-50 days** | **4319** | **639 (14.8%)** | **3680 (85.2%)** |
| **>50 days** | **81** | **8 (9.9%)** | **73 (90.1%)** |
|  |  |  |  |
| **Race** |  |  |  |
| **Asian** | **1543** | **976 (63.3%)** | **567 (36.7%)** |
| **Black/African American** | **4568** | **2767 (60.6%)** | **1801 (39.4%)** |
| **Hispanic/Latino** | **1970** | **1229 (62.4%)** | **741 (37.6%)** |
| **Unknown/Declined/Other** | **9760** | **4976 (51.0%)** | **4784 (49.0%)** |
| **White** | **33997** | **20799 (61.2%)** | **13198 (38.8%)** |
|  |  |  |  |
| **Language** |  |  |  |
| **English** | **46799** | **27841 (59.5%)** | **18958 (40.5%)** |
| **Other** | **5039** | **2906 (57.7%)** | **2133 (42.3%)** |
|  |  |  |  |
| **ICU Type** |  |  |  |
| **Cardiac Vascular Intensive Care Unit (CVICU)** | **10616** | **7092 (66.8%)** | **3524 (33.2%)** |
| **Coronary Care Unit (CCU)** | **5742** | **3853 (67.1%)** | **1889 (32.9%)** |
| **Medical Intensive Care Unit (MICU)** | **9272** | **4772 (51.5%)** | **4500 (48.5%)** |
| **Medical/Surgical Intensive Care Unit (MICU/SICU)** | **7444** | **4734 (63.6%)** | **2710 (36.4%)** |
| **Neuro Intermediate** | **3635** | **2318 (63.8%)** | **1317 (36.2%)** |
| **Neuro Stepdown** | **831** | **466 (56.1%)** | **365 (43.9%)** |
| **Neuro Surgical Intensive Care Unit (Neuro SICU)** | **1091** | **399 (36.6%)** | **692 (63.4%)** |
| **Surgical Intensive Care Unit (SICU)** | **7093** | **3710 (52.3%)** | **3383 (47.7%)** |
| **Trauma SICU (TSICU)** | **5926** | **3362 (56.7%)** | **2564 (43.3%)** |
| **other** | **188** | **41 (21.8%)** | **147 (78.2%)** |
|  |  |  |  |
| **Admission Source** |  |  |  |
| **ED** | **18649** | **10933 (58.6%)** | **7716 (41.4%)** |
| **Other** | **1021** | **653 (64.0%)** | **368 (36.0%)** |
| **Referral** | **16526** | **10628 (64.3%)** | **5898 (35.7%)** |
| **Transfer** | **15642** | **8533 (54.6%)** | **7109 (45.4%)** |
|  |  |  |  |
| **Time Period** |  |  |  |
| **2008 - 2010** | **13627** | **8597 (63.1%)** | **5030 (36.9%)** |
| **2011 - 2013** | **10575** | **6193 (58.6%)** | **4382 (41.4%)** |
| **2014 - 2016** | **10594** | **6094 (57.5%)** | **4500 (42.5%)** |
| **2017 - 2019** | **9874** | **5847 (59.2%)** | **4027 (40.8%)** |
| **2020 - 2022** | **7168** | **4016 (56.0%)** | **3152 (44.0%)** |
|  |  |  |  |
|  |  |  |  |
| **Ventilation** |  |  |  |
| **Invasive Ventilation** | **43412 (83.7%)** | **24116 (78.4%)** | **19296 (91.5%)** |
| **Non-Invasive Ventilation** | **11982 (23.1%)** | **6318 (20.5%)** | **5664 (26.9%)** |
|  |  |  |  |
|  |  |  |  |
| **Lines on the Body** |  |  |  |
| **Central Line** | **1531 (3.0%)** | **556 (1.8%)** | **975 (4.6%)** |
| **Arterial Line** | **5403 (10.4%)** | **2739 (8.9%)** | **2664 (12.6%)** |
| **Dialysis Line** | **6 (0.0%)** | **1 (0.0%)** | **5 (0.0%)** |
|  |  |  |  |
|  |  |  |  |
| **Life Support** |  |  |  |
| **EVD** | **2757 (5.3%)** | **1503 (4.9%)** | **1254 (5.9%)** |
| **ECMO** | **1198 (2.3%)** | **648 (2.1%)** | **550 (2.6%)** |
|  |  |  |  |
|  |  |  |  |
| **Monitoring and Status** |  |  |  |
| **Agitated** | **11943 (23.0%)** | **5117 (16.6%)** | **6826 (32.4%)** |
| **Deeply Sedated** | **14363 (27.7%)** | **6268 (20.4%)** | **8095 (38.4%)** |
| **Chemical Restraint** | **38666 (74.6%)** | **20689 (67.3%)** | **17977 (85.2%)** |
| **Death within 24h of first restraint** | **242 (0.5%)** | **0 (0.0%)** | **242 (1.1%)** |
|  |  |  |  |
|  |  |  |  |
| **Clinical Assessment and Severity Scores** |  |  |  |
| **NRS Recorded** | **37468 (72.3%)** | **24568 (79.9%)** | **12900 (61.2%)** |
| **CPOT Recorded** | **41682 (80.4%)** | **24350 (79.2%)** | **17332 (82.2%)** |
| **CIWA Recorded** | **144 (0.3%)** | **52 (0.2%)** | **92 (0.4%)** |
| **SOFA Score, mean (SD)** | **4.5 (3.4)** | **3.8 (3.0)** | **5.5 (3.6)** |
| **OASIS Score, mean (SD)** | **31.2 (8.5)** | **29.3 (8.1)** | **34.0 (8.3)** |
| **APS-III Score, mean (SD)** | **42.9 (20.3)** | **39.9 (18.5)** | **47.2 (21.9)** |
| **Max NRS Score, mean (SD)** | **4.8 (3.5)** | **5.0 (3.4)** | **4.3 (3.6)** |
| **MAX CPOT Score, mean (SD)** | **1.2 (1.8)** | **0.9 (1.7)** | **1.6 (1.8)** |
| **Max CIWA Score, mean (SD)** | **0.9 (0.3)** | **0.9 (0.3)** | **0.8 (0.4)** |
|  |  |  |  |
|  |  |  |  |
| **Severe Mental Health** |  |  |  |
| **Suicide Attempt** | **312 (0.6%)** | **131 (0.4%)** | **181 (0.9%)** |
| **Psychosis** | **892 (1.7%)** | **332 (1.1%)** | **560 (2.7%)** |
| **Bipolar/Mania** | **1237 (2.4%)** | **581 (1.9%)** | **656 (3.1%)** |
| **Substance Use** | **7201 (13.9%)** | **3143 (10.2%)** | **4058 (19.2%)** |
| **Mental Disorder due to Phys Condition** | **4359 (8.4%)** | **1451 (4.7%)** | **2908 (13.8%)** |
| **Delirious (Positive CAM Score)** | **7986 (15.4%)** | **2296 (7.5%)** | **5690 (27.0%)** |

**Suppl. Table 2** Unadjusted Odds Ratio of Final Model

| **Variable** | **OR (95% CI)** | **p-value** | **Crude OR** | **CI Lower** | **CI Upper** |
| --- | --- | --- | --- | --- | --- |
| **Patient Characteristics** |  |  |  |  |  |
| **Male sex (vs Female)** | **1.16 (1.14 – 1.18)** | **< 0.0001** | **1.1604** | **1.1415843487129000** | **1.179664223005250** |
| **Age (per year)** | **0.99 (0.99 – 0.99)** | **< 0.0001** | **0.9927466464116970** | **0.9922611820688760** | **0.9932323482682210** |
| **Non-English language** | **1.15 (1.12 – 1.18)** | **< 0.0001** | **1.1484579631325500** | **1.119071961625420** | **1.1786156192912100** |
| **Ethnicity: Asian (vs White)** | **0.85 (0.81 – 0.90)** | **< 0.0001** | **0.8544855469762400** | **0.813406736592768** | **0.8976389266822990** |
| **Ethnicity: Hispanic/Latino (vs White)** | **0.93 (0.89 – 0.97)** | **0.0012** | **0.9326279126371820** | **0.893920543218314** | **0.9730113375608650** |
| **Ethnicity: Unknown/Declined/Other (vs White)** | **1.61 (1.58 – 1.64)** | **< 0.0001** | **1.6116114754288700** | **1.5809947430311300** | **1.642821115745410** |
| **Clinical Severity (per point)** |  |  |  |  |  |
| **SOFA score (per point)** | **1.10 (1.10 – 1.11)** | **< 0.0001** | **1.1045534296840100** | **1.1021456552403300** | **1.1069664642108100** |
| **OASIS score (per point)** | **1.06 (1.06 – 1.06)** | **< 0.0001** | **1.0563193090587900** | **1.055289805549620** | **1.057349816915270** |
| **APS III score (per point)** | **1.01 (1.01 – 1.01)** | **< 0.0001** | **1.0128659060994100** | **1.0124999577987900** | **1.0132319866648800** |
| **Respiratory Support & Sedation** |  |  |  |  |  |
| **Invasive ventilation** | **4.84 (4.67 – 5.03)** | **< 0.0001** | **4.8439948156608000** | **4.665196346017390** | **5.029645921372600** |
| **Deep sedation** | **2.25 (2.21 – 2.28)** | **< 0.0001** | **2.2467063352226800** | **2.2091173624607700** | **2.28493489866335** |
| **Chemical Restraint & Delirium** |  |  |  |  |  |
| **Chemical restraint** | **2.88 (2.81 – 2.94)** | **< 0.0001** | **2.8757882337764200** | **2.8093166402063600** | **2.9438326200635700** |
| **Positive CAM screen** | **4.36 (4.27 – 4.46)** | **< 0.0001** | **4.3628051206527400** | **4.272415482705580** | **4.455107092894460** |
| **Invasive Procedures** |  |  |  |  |  |
| **EVD or ECMO** | **1.06 (1.03 – 1.09)** | **0.0001** | **1.058940235234790** | **1.028722043572210** | **1.0900460710506700** |
| **Arterial Line** | **1.25 (1.22 – 1.28)** | **< 0.0001** | **1.2477805681552900** | **1.2175358204916500** | **1.278776624113810** |
| **Central line** | **2.89 (2.77 – 3.00)** | **< 0.0001** | **2.886037711798420** | **2.7732050267588500** | **3.0034611914927000** |
| **Mental Health Diagnoses** |  |  |  |  |  |
| **Psychosis** | **1.80 (1.70 – 1.91)** | **< 0.0001** | **1.8047742432412900** | **1.7040261304022500** | **1.911478944456260** |
| **Mania/Bipolar** | **1.59 (1.51 – 1.67)** | **< 0.0001** | **1.585345815550630** | **1.5065709944572800** | **1.6682395745905700** |
| **Substance use** | **2.33 (2.28 – 2.38)** | **< 0.0001** | **2.333105360914280** | **2.282708962994050** | **2.3846143828985100** |
| **Physical-condition–related mental disorder** | **2.44 (2.38 – 2.50)** | **< 0.0001** | **2.4382290291420400** | **2.377913981082520** | **2.5000739496239400** |
| **ICU Type (vs CVICU)** |  |  |  |  |  |
| **ICU: Cardiac Vascular Intensive Care Unit (CVICU) (vs Cardiac Vascular ICU)** | **0.35 (0.34 – 0.36)** | **< 0.0001** | **0.3479821952819940** | **0.3391520928154200** | **0.35704219669721600** |
| **ICU: Coronary Care Unit (CCU) (vs Cardiac Vascular ICU)** | **0.55 (0.54 – 0.57)** | **< 0.0001** | **0.5523609100748050** | **0.5359899888330430** | **0.5692318538317030** |
| **ICU: Medical Intensive Care Unit (MICU) (vs Cardiac Vascular ICU)** | **1.56 (1.52 – 1.59)** | **< 0.0001** | **1.5556719841470600** | **1.524926789758510** | **1.5870370554925500** |
| **ICU: Medical/Surgical Intensive Care Unit (MICU/SICU) (vs Cardiac Vascular ICU)** | **0.96 (0.94 – 0.98)** | **0.00079** | **0.9592689599211590** | **0.9362539451448280** | **0.9828497302896580** |
| **ICU: Neuro Intermediate (vs Cardiac Vascular ICU)** | **1.29 (1.25 – 1.33)** | **< 0.0001** | **1.2898031044753500** | **1.2547646217378600** | **1.3258200139641700** |
| **ICU: Neuro Stepdown (vs Cardiac Vascular ICU)** | **1.35 (1.27 – 1.43)** | **< 0.0001** | **1.3498437443415300** | **1.2741441419093200** | **1.430040820505250** |
| **ICU: Neuro Surgical Intensive Care Unit (Neuro SICU) (vs Cardiac Vascular ICU)** | **3.31 (3.14 – 3.49)** | **< 0.0001** | **3.3114418350728000** | **3.1405936027006900** | **3.491584208042910** |
| **ICU: Surgical Intensive Care Unit (SICU) (vs Cardiac Vascular ICU)** | **1.21 (1.18 – 1.24)** | **< 0.0001** | **1.2104859740379200** | **1.183641438371730** | **1.2379393335182900** |
| **ICU: Trauma SICU (TSICU) (vs Cardiac Vascular ICU)** | **1.13 (1.10 – 1.15)** | **< 0.0001** | **1.1257663647364300** | **1.0982868084032800** | **1.1539334700872700** |
| **ICU: other (vs Cardiac Vascular ICU)** | **3.53 (3.25 – 3.83)** | **< 0.0001** | **3.5264805158808500** | **3.245962615932860** | **3.8312409292222500** |
| **Time Period (vs 2008–2010)** |  |  |  |  |  |
| **Time Period: 2014–2016 (vs 2008–2010)** | **1.28 (1.26 – 1.31)** | **< 0.0001** | **1.2817682022754400** | **1.256327499965600** | **1.307724080233380** |
| **Time Period: 2017–2019 (vs 2008–2010)** | **1.40 (1.37 – 1.43)** | **< 0.0001** | **1.3982098719505400** | **1.37060259420928** | **1.426373227571340** |
| **Time Period: 2020–2022 (vs 2008–2010)** | **1.64 (1.61 – 1.68)** | **< 0.0001** | **1.6401503555121700** | **1.6056390790427400** | **1.6754034102673300** |

**Primary data analysis model fitting**

Using a base generalized linear model to predict the proportion of restraint days (included the following fixed effects): age, gender, ethnicity, ICU type, ventilation status, deep sedation, chemical restraint, CAM status, and EVD/ECMO status). Following a forward selection approach, we sequentially added blocks of clinically relevant variables. First, several mental health diagnosis flags were added, resulting in a significantly lower (better) AIC. Next, English proficiency (derived from the language variable) was added, again improving the AIC and showing statistical significance. Subsequently, three standardized severity scores (SOFA, OASIS, APSIII) were incorporated simultaneously; this step dramatically lowered the AIC, and all three scores were highly significant (p<0.0001). Following this, the remaining vascular access lines were added, leading to further AIC improvement, although only havin a central line was individually significant. Finally, dummy variables representing 3-year time periods (relative to 2008-2010) were added, again improving the AIC, with most periods showing significance. At this point, the decision was made to stop adding new main effect variables, leaving potential predictors like NIV status, specific RASS/agitation measures, admission path, pain scores, and alcohol withdrawal scores untested.

A backward elimination procedure was performed using a retention criterion of p < 0.05. Starting from the full model with 37 predictors, variables were sequentially removed according to non-significance. The following predictors were excluded in order: suicide attempt diagnosis (p = 0.49), Black/African American ethnicity (p = 0.33), time period 2011–2013 (p = 0.29), and presence of a dialysis line (d-line) (p = 0.23). Each step yielded a small decrease in AIC (total ΔAIC = –4.0), indicating that model parsimony was improved without substantial loss of fit. The final model retained 33 predictors, all of which were statistically significant at p < 0.05.

Throughout model refinement, AIC steadily decreased, indicating improved model fit with successive simplifications. Although the Hosmer–Lemeshow goodness-of-fit test returned a statistically significant result (χ² = 455.6, df = 8, p < 0.0001), suggesting poor calibration, graphical assessment demonstrated that the final simplified model predicted restraint-day proportions accurately across most of the probability spectrum. In the lower predicted-risk deciles (0.0–0.4), observed and expected restraint proportions were nearly identical, indicating good fit for the majority of patients. Mid-range predictions (0.4–0.6) showed only minor systematic deviation from the ideal 45° line, and overestimation was limited to the highest-risk deciles (0.6–0.8), likely reflecting residual confounding or unit-level effects not explicitly modelled. This divergence between strong visual alignment and a significant Hosmer–Lemeshow p-value is a common artifact of very large samples, where even trivial departures from perfect calibration become statistically detectable. The calibration slope (0.94) and intercept (–0.03) further confirmed good overall agreement between predicted and observed outcomes. Multicollinearity was minimal (all variance-inflation factors < 5 except the model constant, VIF = 30.7), indicating no meaningful redundancy among predictors. Collectively, these findings support the adequacy and stability of the final 33-variable model for assessing demographic and clinical disparities in restraint utilization.

**Secondary Outcome Analyses**

**Suppl. Table 3** Binary Yes/No Restraint

| **Variable** | **Coef** | **SE** | **OR** | **CI_Lower** | **CI_Upper** | **P_value** | **Sig** | **Category** |
| --- | --- | --- | --- | --- | --- | --- | --- | --- |
| **const** | -3.792993302983060 | 0.05941295309491560 | 0.02252806749451730 | 0.020051715831418400 | 0.025310244235673800 | < 0.0001 | *** | 0_Intercept |
| **race_Unknown_Declined_Other** | 0.147531162865381 | 0.02669940450916360 | 1.1589694010744300 | 1.0998802295167900 | 1.2212330366343100 | < 0.0001 | *** | 1_Demographics |
| **race_Hispanic_Latino** | -0.11547569289796700 | 0.0576973699616256 | 0.8909422281975260 | 0.7956781890266410 | 0.9976119302159180 | 0.045 | * | 1_Demographics |
| **race_Asian** | -0.05580509347170140 | 0.0633528044411012 | 0.9457234456017430 | 0.8352916710350230 | 1.0707551225221400 | 0.37 |  | 1_Demographics |
| **physical_condition_mental_disorder** | 0.9089074555275800 | 0.037640581578014800 | 2.481609782703480 | 2.3051212342687700 | 2.6716109426509900 | < 0.0001 | *** | 2_Mental_Health |
| **substance_use_diagnosis** | 0.543733344001317 | 0.030472328272611800 | 1.7224252797354300 | 1.6225657263170000 | 1.8284306121797500 | < 0.0001 | *** | 2_Mental_Health |
| **psychosis_diagnosis** | 0.5468985732884580 | 0.07814771347994130 | 1.7278857879971800 | 1.4825029909525100 | 2.0138841638655800 | < 0.0001 | *** | 2_Mental_Health |
| **mania_bipolar_diagnosis** | 0.1746413644589290 | 0.06551448216228290 | 1.190819069980480 | 1.0473208573527700 | 1.353978627918730 | 0.0076 | ** | 2_Mental_Health |
| **positive_cam_day1** | 0.9981058280719540 | 0.02968268598443270 | 2.713137808693990 | 2.559799237534250 | 2.8756617554334100 | < 0.0001 | *** | 3_Clinical_Status |
| **chemical_restraint_day1** | 0.6662164785583090 | 0.02755615681403000 | 1.9468573916945100 | 1.8444984609422400 | 2.054896647438440 | < 0.0001 | *** | 3_Clinical_Status |
| **deeply_sedated_day1** | 0.5830227528119780 | 0.027606932614358000 | 1.791445351214010 | 1.6970885397415400 | 1.8910483284950100 | < 0.0001 | *** | 3_Clinical_Status |
| **first_day_oasis** | 0.037315337278169500 | 0.0017264226206365800 | 1.0380202957292000 | 1.0345138550645500 | 1.0415386213251800 | < 0.0001 | *** | 4_Severity_Scores |
| **first_day_sofa** | 0.05984285390572330 | 0.0046375987904510600 | 1.061669696189590 | 1.0520633459685600 | 1.0713637616275100 | < 0.0001 | *** | 4_Severity_Scores |
| **first_day_apsiii** | -0.004486175916790290 | 0.0007939037469621910 | 0.995523871939289 | 0.9939760187048820 | 0.9970741355433530 | < 0.0001 | *** | 4_Severity_Scores |
| **icu_type_Surgical_Intensive_Care_Unit_SICU** | 1.1499094924148600 | 0.03835187827242370 | 3.157907082211080 | 2.9292346938162500 | 3.404430911913970 | < 0.0001 | *** | 5_ICU_Type |
| **icu_type_Medical_Intensive_Care_Unit_MICU** | 0.8324517092196750 | 0.03744894218106580 | 2.298948189029230 | 2.136252511885400 | 2.474034668858620 | < 0.0001 | *** | 5_ICU_Type |
| **icu_type_Neuro_Intermediate** | 1.077936997349790 | 0.05072210867841090 | 2.9386109312893200 | 2.6605250515612400 | 3.2457631625853900 | < 0.0001 | *** | 5_ICU_Type |
| **icu_type_Neuro_Surgical_Intensive_Care_Unit_Neuro_SICU** | 1.4974795898963000 | 0.0763629329638097 | 4.470407598862990 | 3.848990828736040 | 5.192151654602570 | < 0.0001 | *** | 5_ICU_Type |
| **icu_type_Trauma_SICU_TSICU** | 0.7114258079683350 | 0.04054761934885350 | 2.036893408053070 | 1.8812828937005600 | 2.2053752626267300 | < 0.0001 | *** | 5_ICU_Type |
| **icu_type_Neuro_Stepdown** | 1.3905116443662800 | 0.08501656136422770 | 4.016904753998560 | 3.400363188291390 | 4.74523540845766 | < 0.0001 | *** | 5_ICU_Type |
| **icu_type_Medical_Surgical_Intensive_Care_Unit_MICU_SICU** | 0.6304565878138930 | 0.04015403772290050 | 1.8784680691188800 | 1.7362994924365100 | 2.0322774395030000 | < 0.0001 | *** | 5_ICU_Type |
| **icu_type_Coronary_Care_Unit_CCU** | 0.6245964724882530 | 0.04199614840964220 | 1.8674922208766300 | 1.7199333397515900 | 2.027710676007040 | < 0.0001 | *** | 5_ICU_Type |
| **icu_type_other** | 1.9741138928631400 | 0.19785637958439600 | 7.20023664514502 | 4.885748582342140 | 10.611149319770400 | < 0.0001 | *** | 5_ICU_Type |
| **time_period_2017 - 2019** | -0.2633506015873470 | 0.0307663297541065 | 0.7684724224236250 | 0.7235023391290000 | 0.8162376706847650 | < 0.0001 | *** | 6_Time_Period |
| **time_period_2014 - 2016** | -0.14229001019871400 | 0.027477055284095500 | 0.8673696739554250 | 0.8218938332013490 | 0.9153617181517820 | < 0.0001 | *** | 6_Time_Period |
| **time_period_2020 - 2022** | -0.1171220280894270 | 0.03456658419059470 | 0.88947664540498 | 0.831211263688759 | 0.9518262531836240 | 0.0007 | *** | 6_Time_Period |
| **ventilated_day1** | 0.4886295382175970 | 0.03278246855766580 | 1.6300807251391200 | 1.5286379046958000 | 1.7382554510179200 | < 0.0001 | *** | 7_Other |
| **gender_M** | 0.13946214236401000 | 0.02077159045964980 | 1.1496552816630400 | 1.1037909501334600 | 1.197425351689930 | < 0.0001 | *** | 7_Other |
| **evd_or_ecmo** | 0.1723056868663490 | 0.03888718835450490 | 1.1880409462255800 | 1.1008561532782100 | 1.2821305360427800 | < 0.0001 | *** | 7_Other |
| **has_aline_day1** | 0.1416397386222500 | 0.03317869965018480 | 1.152161494472120 | 1.0796216786853100 | 1.2295752628464700 | < 0.0001 | *** | 7_Other |
| **age** | -0.03869026014250750 | 0.011415850847525400 | 0.9620486478133190 | 0.9407621666592430 | 0.9838167749093580 | 0.0007 | *** | 7_Other |
| **non_english_language** | 0.11569211609231400 | 0.03811140143187660 | 1.1226501729961600 | 1.0418470441209400 | 1.2097201964917200 | 0.0024 | ** | 7_Other |
| **has_cline_day1** | 0.18619094205247700 | 0.06390242804357630 | 1.204652257193410 | 1.0628399085267300 | 1.3653863099408400 | 0.0035 | ** | 7_Other |

**Suppl. Table 4** Mortality within 24 Hours of First Restraint

| **Variable** | **Coef** | **SE** | **OR** | **CI_Lower** | **CI_Upper** | **P_value** | **Sig** | **Category** |
| --- | --- | --- | --- | --- | --- | --- | --- | --- |
| **const** | -8.183146362676630 | 0.5336389576952300 | 0.0002793217086922010 | 9.81454986237674E-05 | 0.0007949485003465770 | < 0.0001 | *** | 0_Intercept |
| **race_Unknown_Declined_Other** | 0.1570699912372810 | 0.15860907082137900 | 1.1700775062073800 | 0.8574451429762240 | 1.596698496396340 | 0.32 |  | 1_Demographics |
| **race_Asian** | 0.16378552752734800 | 0.4250320075858560 | 1.1779616476513200 | 0.5120864095051560 | 2.709686524737680 | 0.69 |  | 1_Demographics |
| **race_Hispanic_Latino** | 0.07065180857766560 | 0.42262626630866000 | 1.0732074791658000 | 0.4687524606578220 | 2.4571055941148200 | 0.86 |  | 1_Demographics |
| **physical_condition_mental_disorder** | -1.1112335309061000 | 0.282409975802833 | 0.3291526905364690 | 0.1892385458598250 | 0.5725128207635290 | < 0.0001 | *** | 2_Mental_Health |
| **psychosis_diagnosis** | -0.7028199169125640 | 0.60485930501444 | 0.49518694704627100 | 0.15132546407659400 | 1.6204154008137900 | 0.24 |  | 2_Mental_Health |
| **mania_bipolar_diagnosis** | -0.5126251236273280 | 0.5925816999677470 | 0.5989212709606710 | 0.18748359823805500 | 1.9132697056181000 | 0.38 |  | 2_Mental_Health |
| **substance_use_diagnosis** | 0.07590580544798370 | 0.1920831961607800 | 1.0788609465474300 | 0.7403958220977030 | 1.572052282369060 | 0.69 |  | 2_Mental_Health |
| **positive_cam_day1** | -0.48491851997837600 | 0.16439450941112400 | 0.615747365829052 | 0.4461384997198930 | 0.849836583848876 | 0.0031 | ** | 3_Clinical_Status |
| **chemical_restraint_day1** | -0.43659747008117000 | 0.21174644665887000 | 0.6462315065789830 | 0.4267261188508850 | 0.9786491654645450 | 0.039 | * | 3_Clinical_Status |
| **deeply_sedated_day1** | 0.322541669755034 | 0.16298226474222200 | 1.3806324202756900 | 1.0031071178565400 | 1.90024160529276 | 0.047 | * | 3_Clinical_Status |
| **first_day_apsiii** | 0.026401863506597600 | 0.004136026302244440 | 1.026753480331160 | 1.0184637870924200 | 1.0351106467730300 | < 0.0001 | *** | 4_Severity_Scores |
| **first_day_sofa** | 0.07429588137661500 | 0.02505273163375890 | 1.0771254597157800 | 1.0255134660620200 | 1.1313349793670600 | 0.0030 | ** | 4_Severity_Scores |
| **first_day_oasis** | 0.0010037706137176100 | 0.01155453678820610 | 1.0010042745600400 | 0.978589817502633 | 1.0239321314875400 | 0.93 |  | 4_Severity_Scores |
| **icu_type_Neuro_Surgical_Intensive_Care_Unit_Neuro_SICU** | 2.9256946459509900 | 0.4873350565124900 | 18.647174736430100 | 7.174517319094570 | 48.465577569313000 | < 0.0001 | *** | 5_ICU_Type |
| **icu_type_Medical_Intensive_Care_Unit_MICU** | 2.0791873425821300 | 0.41035664222675000 | 7.997966665665190 | 3.5783546614740300 | 17.876224364731200 | < 0.0001 | *** | 5_ICU_Type |
| **icu_type_Medical_Surgical_Intensive_Care_Unit_MICU_SICU** | 2.0452681949621400 | 0.4232819878953610 | 7.731231737289710 | 3.372487974640650 | 17.723397273801900 | < 0.0001 | *** | 5_ICU_Type |
| **icu_type_Coronary_Care_Unit_CCU** | 2.053128929530680 | 0.42560028078467000 | 7.792244385812300 | 3.383692968850430 | 17.944616467034800 | < 0.0001 | *** | 5_ICU_Type |
| **icu_type_Surgical_Intensive_Care_Unit_SICU** | 1.9171044868720300 | 0.4242049890111800 | 6.8012368603652100 | 2.9614469588639800 | 15.619669530915600 | < 0.0001 | *** | 5_ICU_Type |
| **icu_type_Trauma_SICU_TSICU** | 1.677045937981910 | 0.45356711459084300 | 5.349729174200920 | 2.1991495749964000 | 13.013940735406 | 0.0002 | *** | 5_ICU_Type |
| **icu_type_Neuro_Intermediate** | -20.277835771490800 | 15534.612022293200 | 1.56116145309159E-09 | 0.0 | inf | 0.99 |  | 5_ICU_Type |
| **icu_type_Neuro_Stepdown** | -20.280099167117800 | 29658.94822249770 | 1.55763192295401E-09 | 0.0 | inf | 0.99 |  | 5_ICU_Type |
| **icu_type_other** | -21.199490398392400 | 45540.68133260290 | 6.21123986084863E-10 | 0.0 | inf | 0.99 |  | 5_ICU_Type |
| **time_period_2020 - 2022** | -0.1669721399942630 | 0.23027095315959600 | 0.8462231867916200 | 0.538862461086173 | 1.3288988073512300 | 0.46 |  | 6_Time_Period |
| **time_period_2014 - 2016** | 0.11750387866612800 | 0.17962995092620200 | 1.1246859922167400 | 0.7909153323484440 | 1.5993097229922900 | 0.51 |  | 6_Time_Period |
| **time_period_2017 - 2019** | 0.04909839354900550 | 0.20439877244530400 | 1.0503236907314900 | 0.7036207052760560 | 1.5678615581373400 | 0.81 |  | 6_Time_Period |
| **age** | 0.5043349407479300 | 0.0878771283730277 | 1.6558838932742000 | 1.3938907449783900 | 1.96712079327813 | < 0.0001 | *** | 7_Other |
| **evd_or_ecmo** | 0.5041062679125250 | 0.2154940874522820 | 1.6555052809000600 | 1.085179724119550 | 2.525570349475180 | 0.019 | * | 7_Other |
| **has_cline_day1** | 0.3632094216877920 | 0.21005885176856300 | 1.437936962897 | 0.9526588993669310 | 2.170412422158100 | 0.083 |  | 7_Other |
| **has_aline_day1** | 0.13632152048865100 | 0.18125990826714400 | 1.1460503130211500 | 0.803368817036658 | 1.6349045321682500 | 0.45 |  | 7_Other |
| **non_english_language** | -0.14592319644259100 | 0.2448891990750500 | 0.8642240761192160 | 0.534781403681106 | 1.3966141092473100 | 0.55 |  | 7_Other |
| **gender_M** | -0.0477269952620686 | 0.13635337505920300 | 0.9533940326397790 | 0.7298073384905870 | 1.2454796403569800 | 0.72 |  | 7_Other |
| **ventilated_day1** | 0.04936724397373420 | 0.27326512925262400 | 1.0506061086642300 | 0.614944925270054 | 1.7949139023756900 | 0.85 |  | 7_Other |

**Suppl. Table 5** Final model stratified by ICU type

|  | **Cardiac Vascular Intensive Care Unit (CVICU)** | **Coronary Care Unit (CCU)** | **Medical Intensive Care Unit (MICU)** | **Medical/Surgical Intensive Care Unit (MICU/SICU)** | **Neuro Intermediate** | **Neuro Stepdown** | **Neuro Surgical Intensive Care Unit (Neuro SICU)** | **Surgical Intensive Care Unit (SICU)** | **Trauma SICU (TSICU)** | **other** |
| --- | --- | --- | --- | --- | --- | --- | --- | --- | --- | --- |
| **Race: Asian** | 0.61 (0.50–0.75)*** | 0.61 (0.47–0.79)*** | 0.79 (0.69–0.90)*** | 0.83 (0.72–0.97)* | 1.08 (0.89–1.31) | 0.58 (0.38–0.91)* | 1.57 (1.13–2.17)** | 0.83 (0.73–0.95)** | 0.99 (0.82–1.20) | 0.63 (0.37–1.10) |
| **Race: Hispanic/Latino** | 0.83 (0.72–0.97)* | 0.73 (0.59–0.90)** | 0.85 (0.76–0.95)** | 0.78 (0.68–0.91)** | 0.72 (0.59–0.87)*** | 0.72 (0.45–1.17) | 1.96 (1.40–2.75)*** | 1.01 (0.89–1.15) | 0.97 (0.84–1.12) | 0.90 (0.57–1.40) |
| **Race: Unknown/Other** | 1.02 (0.95–1.08) | 1.01 (0.94–1.10) | 0.96 (0.92–1.01) | 1.22 (1.13–1.31)*** | 1.39 (1.28–1.50)*** | 0.88 (0.73–1.07) | 1.53 (1.34–1.75)*** | 1.02 (0.96–1.08) | 0.98 (0.92–1.04) | 0.80 (0.64–1.01) |
| **Male gender** | 1.17 (1.11–1.24)*** | 1.21 (1.13–1.29)*** | 1.16 (1.12–1.21)*** | 1.28 (1.21–1.35)*** | 1.49 (1.40–1.60)*** | 1.34 (1.16–1.55)*** | 1.03 (0.91–1.16) | 1.10 (1.05–1.16)*** | 1.22 (1.15–1.29)*** | 1.26 (1.02–1.55)* |
| **Non-English language** | 1.37 (1.24–1.50)*** | 1.29 (1.15–1.46)*** | 1.27 (1.18–1.36)*** | 1.45 (1.32–1.59)*** | 1.32 (1.18–1.47)*** | 1.41 (1.11–1.79)** | 0.89 (0.73–1.07) | 1.15 (1.05–1.25)** | 0.89 (0.81–0.99)* | 0.50 (0.37–0.66)*** |
| **Psychosis diagnosis** | 1.63 (1.32–2.01)*** | 1.28 (0.98–1.69) | 1.08 (0.96–1.21) | 1.38 (1.18–1.62)*** | 0.62 (0.44–0.86)** | 1.50 (0.82–2.75) | 2.04 (1.28–3.26)** | 1.59 (1.35–1.87)*** | 1.52 (1.25–1.86)*** | 1.30 (0.82–2.04) |
| **Mania/Bipolar diagnosis** | 1.21 (0.95–1.54) | 1.12 (0.89–1.40) | 1.36 (1.22–1.51)*** | 1.28 (1.10–1.48)** | 1.06 (0.84–1.34) | 0.64 (0.31–1.34) | 0.88 (0.59–1.30) | 0.97 (0.81–1.15) | 0.64 (0.56–0.74)*** | 0.79 (0.40–1.55) |
| **Mental disorder due to physical condition** | 2.99 (2.78–3.21)*** | 2.04 (1.85–2.24)*** | 1.54 (1.45–1.64)*** | 1.65 (1.52–1.79)*** | 2.43 (2.20–2.69)*** | 2.15 (1.64–2.80)*** | 1.72 (1.41–2.10)*** | 1.89 (1.75–2.05)*** | 1.91 (1.76–2.06)*** | 2.06 (1.58–2.67)*** |
| **Substance-use diagnosis** | 1.42 (1.31–1.54)*** | 1.80 (1.63–2.00)*** | 1.70 (1.62–1.80)*** | 1.68 (1.56–1.82)*** | 1.47 (1.34–1.60)*** | 1.08 (0.90–1.31) | 1.81 (1.55–2.11)*** | 1.54 (1.44–1.66)*** | 2.08 (1.94–2.24)*** | 1.09 (0.82–1.46) |
| **Ventilated (day 1)** | 1.26 (1.07–1.49)** | 1.54 (1.40–1.69)*** | 2.06 (1.89–2.24)*** | 2.66 (2.44–2.90)*** | 2.97 (2.28–3.87)*** | 5.20 (2.89–9.38)*** | 4.49 (2.76–7.30)*** | 1.76 (1.59–1.94)*** | 2.64 (2.17–3.22)*** | 10672396596.73 (0.00–inf) |
| **Deep sedation (day 1)** | 1.25 (1.18–1.33)*** | 1.89 (1.74–2.06)*** | 1.75 (1.67–1.84)*** | 2.23 (2.08–2.39)*** | 2.50 (2.22–2.81)*** | 1.63 (1.34–1.99)*** | 1.43 (1.25–1.65)*** | 1.78 (1.69–1.88)*** | 1.32 (1.25–1.41)*** | 1.07 (0.76–1.49) |
| **Chemical restraint (day 1)** | 1.27 (1.10–1.46)*** | 1.71 (1.56–1.87)*** | 1.95 (1.84–2.08)*** | 1.56 (1.45–1.68)*** | 3.58 (3.33–3.85)*** | 2.44 (2.05–2.91)*** | 2.82 (2.43–3.28)*** | 1.69 (1.57–1.81)*** | 2.56 (2.32–2.83)*** | 4.70 (2.99–7.38)*** |
| **Positive CAM (day 1)** | 1.44 (1.33–1.57)*** | 2.31 (2.12–2.51)*** | 2.40 (2.29–2.51)*** | 2.41 (2.25–2.58)*** | 3.52 (3.26–3.81)*** | 3.20 (2.72–3.76)*** | 2.73 (2.41–3.11)*** | 2.46 (2.31–2.62)*** | 2.41 (2.25–2.58)*** | 4.38 (3.28–5.87)*** |
| **Central line (day 1)** | 1.64 (1.28–2.10)*** | 1.60 (1.33–1.92)*** | 1.15 (1.07–1.24)*** | 1.09 (0.95–1.25) | 0.16 (0.06–0.44)*** | 11.06 (1.50–81.77)* | 3.16 (2.15–4.63)*** | 1.34 (1.18–1.53)*** | 1.38 (1.23–1.54)*** | 1.78 (1.22–2.60)** |
| **Arterial line (day 1)** | 1.15 (1.05–1.25)** | 1.21 (1.09–1.34)*** | 0.95 (0.89–1.01) | 1.04 (0.95–1.13) | 0.57 (0.48–0.67)*** | 1.00 (0.73–1.38) | 0.68 (0.53–0.87)** | 0.94 (0.88–1.01) | 1.23 (1.17–1.31)*** | 0.31 (0.21–0.44)*** |
| **SOFA score (per point)** | 1.01 (1.00–1.02) | 1.02 (1.01–1.04)** | 1.04 (1.04–1.05)*** | 1.04 (1.03–1.05)*** | 1.16 (1.13–1.19)*** | 1.16 (1.10–1.21)*** | 0.99 (0.96–1.02) | 1.04 (1.03–1.05)*** | 1.06 (1.05–1.07)*** | 1.04 (1.00–1.08)* |
| **OASIS score (per point)** | 1.02 (1.01–1.02)*** | 1.03 (1.02–1.03)*** | 1.03 (1.02–1.03)*** | 1.03 (1.03–1.04)*** | 1.04 (1.04–1.05)*** | 1.07 (1.06–1.09)*** | 1.02 (1.01–1.03)*** | 1.03 (1.03–1.03)*** | 1.04 (1.03–1.04)*** | 0.93 (0.91–0.95)*** |
| **APS III (per point)** | 1.01 (1.00–1.01)*** | 1.00 (1.00–1.00) | 0.99 (0.99–0.99)*** | 0.99 (0.99–1.00)*** | 0.99 (0.99–1.00)*** | 0.99 (0.98–0.99)** | 1.01 (1.00–1.01)* | 0.99 (0.99–1.00)*** | 0.99 (0.99–0.99)*** | 1.00 (0.99–1.01) |
| **Age (per decade)** | 0.93 (0.89–0.96)*** | 1.07 (1.02–1.11)** | 0.94 (0.92–0.96)*** | 0.86 (0.83–0.88)*** | 0.96 (0.92–1.00)* | 0.92 (0.84–1.00)* | 1.06 (1.00–1.13) | 0.90 (0.88–0.93)*** | 0.91 (0.89–0.93)*** | 1.27 (1.12–1.45)*** |
| **Period 2017–2019** | 0.62 (0.57–0.67)*** | 0.78 (0.71–0.86)*** | 1.64 (1.54–1.74)*** | 1.27 (1.17–1.37)*** | 0.89 (0.80–1.00) | 0.90 (0.70–1.15) | 0.77 (0.61–0.96)* | 1.94 (1.79–2.11)*** | 3.06 (2.83–3.30)*** | 1.32 (0.86–2.02) |
| **Period 2020–2022** | 0.73 (0.67–0.79)*** | 1.37 (1.24–1.52)*** | 1.59 (1.49–1.70)*** | 1.71 (1.57–1.86)*** | 0.83 (0.74–0.94)** |  | 0.98 (0.78–1.24) | 1.88 (1.72–2.05)*** | 3.51 (3.22–3.83)*** | 1.19 (0.87–1.63) |
| **McFadden Pseudo-R²** | 0.077 | 0.198 | 0.216 | 0.243 | 0.378 | 0.365 | 0.198 | 0.216 | 0.226 | 0.274 |

**Suppl. Table 6** Matched Cohorts vs Unmatched

| **Variable** | **Main PSM** | **No Demographics PSM** | **No Demo + No Psych PSM** | **Unmatched** |
| --- | --- | --- | --- | --- |
| **N** | **4025** | **4205** | **4199** | **23409** |
| **age** | **59.77 ± 17.43*** | **64.57 ± 16.66*** | **65.09 ± 16.60** | **66.33 ± 15.34** |
| **gender** |  |  |  |  |
| **F** | **2028 (50.4%)*** | **1850 (44.0%)*** | **1873 (44.6%)*** | **9692 (41.4%)** |
| **M** | **1997 (49.6%)*** | **2355 (56.0%)*** | **2326 (55.4%)*** | **13717 (58.6%)** |
| **language** |  |  |  |  |
| **English** | **3781 (93.9%)*** | **4010 (95.4%)*** | **4035 (96.1%)** | **22710 (97.0%)** |
| **Other** | **244 (6.1%)*** | **195 (4.6%)*** | **164 (3.9%)** | **699 (3.0%)** |
| **icu_type** |  |  |  |  |
| **Cardiac Vascular Intensive Care Unit (CVICU)** | **386 (9.6%)*** | **417 (9.9%)*** | **414 (9.9%)*** | **6413 (27.4%)** |
| **Coronary Care Unit (CCU)** | **386 (9.6%)*** | **412 (9.8%)*** | **491 (11.7%)** | **2710 (11.6%)** |
| **Medical Intensive Care Unit (MICU)** | **989 (24.6%)*** | **1047 (24.9%)*** | **1037 (24.7%)*** | **3352 (14.3%)** |
| **Medical/Surgical Intensive Care Unit (MICU/SICU)** | **793 (19.7%)*** | **858 (20.4%)*** | **829 (19.7%)*** | **2821 (12.1%)** |
| **Neuro Intermediate** | **336 (8.3%)*** | **347 (8.3%)*** | **340 (8.1%)*** | **1393 (6.0%)** |
| **Neuro Stepdown** | **69 (1.7%)** | **71 (1.7%)** | **71 (1.7%)** | **359 (1.5%)** |
| **Neuro Surgical Intensive Care Unit (Neuro SICU)** | **85 (2.1%)** | **75 (1.8%)** | **63 (1.5%)** | **306 (1.3%)** |
| **Surgical Intensive Care Unit (SICU)** | **598 (14.9%)** | **599 (14.2%)** | **598 (14.2%)** | **3249 (13.9%)** |
| **Trauma SICU (TSICU)** | **370 (9.2%)*** | **376 (8.9%)*** | **351 (8.4%)*** | **2750 (11.7%)** |
| **other** | **13 (0.3%)** | **3 (0.1%)** | **5 (0.1%)** | **56 (0.2%)** |
| **admission_source** |  |  |  |  |
| **ED** | **1924 (47.8%)*** | **2024 (48.1%)*** | **2034 (48.4%)*** | **7500 (32.0%)** |
| **Other** | **57 (1.4%)** | **60 (1.4%)** | **55 (1.3%)*** | **548 (2.3%)** |
| **Referral** | **1306 (32.4%)** | **1343 (31.9%)*** | **1307 (31.1%)*** | **7961 (34.0%)** |
| **Transfer** | **738 (18.3%)*** | **778 (18.5%)*** | **803 (19.1%)*** | **7400 (31.6%)** |
| **time_period** |  |  |  |  |
| **2008 - 2010** | **1403 (34.9%)*** | **1477 (35.1%)*** | **1472 (35.1%)*** | **6311 (27.0%)** |
| **2011 - 2013** | **898 (22.3%)*** | **915 (21.8%)*** | **943 (22.5%)*** | **4778 (20.4%)** |
| **2014 - 2016** | **689 (17.1%)*** | **722 (17.2%)*** | **721 (17.2%)*** | **5012 (21.4%)** |
| **2017 - 2019** | **635 (15.8%)*** | **674 (16.0%)*** | **656 (15.6%)*** | **4368 (18.7%)** |
| **2020 - 2022** | **400 (9.9%)*** | **417 (9.9%)*** | **407 (9.7%)*** | **2940 (12.6%)** |
| **ventilated_day1** | **0.79 ± 0.41*** | **0.79 ± 0.41*** | **0.79 ± 0.41*** | **0.84 ± 0.37** |
| **deeply_sedated_day1** | **0.22 ± 0.42*** | **0.21 ± 0.41*** | **0.21 ± 0.41*** | **0.27 ± 0.44** |
| **positive_cam_day1** | **0.15 ± 0.36** | **0.15 ± 0.36** | **0.15 ± 0.36** | **0.14 ± 0.34** |
| **chemical_restraint_day1** | **0.68 ± 0.47*** | **0.68 ± 0.46*** | **0.68 ± 0.46*** | **0.78 ± 0.42** |
| **has_cline_day1** | **0.04 ± 0.19** | **0.03 ± 0.17** | **0.03 ± 0.18** | **0.02 ± 0.15** |
| **evd_or_ecmo** | **0.05 ± 0.22*** | **0.06 ± 0.23*** | **0.06 ± 0.23*** | **0.09 ± 0.28** |
| **first_day_sofa** | **4.36 ± 3.47** | **4.34 ± 3.34** | **4.38 ± 3.36** | **4.41 ± 3.20** |
| **first_day_oasis** | **30.42 ± 8.73** | **30.58 ± 8.61** | **30.83 ± 8.56** | **31.21 ± 8.29** |
| **first_day_apsiii** | **43.25 ± 20.86** | **43.74 ± 20.02** | **43.44 ± 20.01** | **42.15 ± 19.36** |
| **psychosis_diagnosis** | **0.03 ± 0.16*** | **0.01 ± 0.12** | **0.02 ± 0.13** | **0.01 ± 0.11** |
| **mania_bipolar_diagnosis** | **0.02 ± 0.15** | **0.02 ± 0.13** | **0.03 ± 0.16** | **0.02 ± 0.15** |
| **substance_use_diagnosis** | **0.14 ± 0.35** | **0.13 ± 0.33** | **0.13 ± 0.34** | **0.13 ± 0.34** |
| **physical_condition_mental_disorder** | **0.08 ± 0.27** | **0.08 ± 0.28** | **0.08 ± 0.28** | **0.09 ± 0.28** |
| **length_of_stay** | **4.74 ± 5.39** | **4.63 ± 5.27** | **4.74 ± 5.60** | **4.49 ± 5.05** |

Notes: Values in each matched cohort are compared with the Unmatched White cohort (reference). Asterisks (*) denote differences surviving Benjamini–Hochberg false discovery rate (q<0.05) within variable and passing a minimal effect-size guard (continuous: |Cohen’s d| ≥ 0.10; categorical: |Δ percentage points| ≥ 1.0). Matched White cohorts represent the subset of White patients selected to resemble Black patients on observed covariates; they are not representative of all White patients.

**Suppl. Table 7** Propensity Score Matching

| **Variable** | **Full PSM OR** | **Full PSM CI_Lower** | **Full PSM CI_Upper** | **Full PSM p** | **No Demographics OR** | **No Demographics CI_Lower** | **No Demographics CI_Upper** | **No Demographics p** | **No Demographics + Psych OR** | **No Demographics + Psych CI_Lower** | **No Demographics + Psych CI_Upper** | **No Demographics + Psych p** |
| --- | --- | --- | --- | --- | --- | --- | --- | --- | --- | --- | --- | --- |
| **black_ethn** | 1.009839 | 0.963206 | 1.05873 | 0.68 | 1.141579 | 1.088174 | 1.197605 | < 0.0001 | 1.011686 | 0.96438 | 1.061313 | 0.63 |
| **positive_cam_day1** | 2.710816 | 2.55403 | 2.877226 | < 0.0001 | 2.545166 | 2.397777 | 2.701614 | < 0.0001 | 2.690086 | 2.535655 | 2.853923 | < 0.0001 |
| **chemical_restraint_day1** | 2.406072 | 2.244109 | 2.579724 | < 0.0001 | 2.148226 | 2.00613 | 2.300387 | < 0.0001 | 2.35894 | 2.201444 | 2.527703 | < 0.0001 |
| **deeply_sedated_day1** | 1.725006 | 1.625316 | 1.830811 | < 0.0001 | 1.810578 | 1.706906 | 1.920548 | < 0.0001 | 1.878358 | 1.772931 | 1.990055 | < 0.0001 |
| **physical_condition_mental_disorder** | 1.966073 | 1.820425 | 2.123375 | < 0.0001 | 1.932616 | 1.795116 | 2.080647 | < 0.0001 | 2.180996 | 2.028282 | 2.345208 | < 0.0001 |
| **evd_or_ecmo** | 0.9614 | 0.867431 | 1.065548 | 0.45 | 1.022494 | 0.927422 | 1.127311 | 0.66 | 0.927746 | 0.842197 | 1.021984 | 0.13 |
| **const** | 0.008697 | 0.007371 | 0.010263 | < 0.0001 | 0.006882 | 0.005838 | 0.008113 | < 0.0001 | 0.009153 | 0.007793 | 0.01075 | < 0.0001 |
| **age** | 0.933974 | 0.908266 | 0.960411 | < 0.0001 | 0.997007 | 0.971035 | 1.023674 | 0.82 | 0.930473 | 0.90668 | 0.954891 | < 0.0001 |
| **first_day_sofa** | 1.057489 | 1.046854 | 1.068232 | < 0.0001 | 1.051317 | 1.040988 | 1.061748 | < 0.0001 | 1.054257 | 1.044065 | 1.064549 | < 0.0001 |
| **gender_M** | 1.125847 | 1.072876 | 1.181434 | < 0.0001 | 1.276869 | 1.217317 | 1.339335 | < 0.0001 | 1.223826 | 1.167272 | 1.283119 | < 0.0001 |
| **has_aline_day1** | 1.000298 | 0.923822 | 1.083106 | 0.99 | 0.928373 | 0.860795 | 1.001256 | 0.05 | 0.956065 | 0.88287 | 1.03533 | 0.27 |
| **has_cline_day1** | 1.089563 | 0.980691 | 1.210522 | 0.11 | 1.140982 | 1.014759 | 1.282905 | 0.027 | 1.164664 | 1.041581 | 1.302292 | 0.0075 |
| **icu_type_Coronary_Care_Unit_CCU** | 2.316112 | 2.032691 | 2.639051 | < 0.0001 | 2.055727 | 1.803683 | 2.342991 | < 0.0001 | 2.121032 | 1.869092 | 2.406931 | < 0.0001 |
| **icu_type_Medical_Intensive_Care_Unit_MICU** | 3.726198 | 3.350549 | 4.143963 | < 0.0001 | 3.976257 | 3.576901 | 4.420201 | < 0.0001 | 3.386026 | 3.053383 | 3.754908 | < 0.0001 |
| **first_day_apsiii** | 0.995714 | 0.994031 | 0.9974 | < 0.0001 | 0.998262 | 0.996568 | 0.999958 | 0.045 | 0.994928 | 0.993256 | 0.996602 | < 0.0001 |
| **first_day_oasis** | 1.019762 | 1.015643 | 1.023898 | < 0.0001 | 1.023235 | 1.019131 | 1.027356 | < 0.0001 | 1.02284 | 1.018772 | 1.026925 | < 0.0001 |
| **icu_type_Neuro_Intermediate** | 5.791266 | 5.109004 | 6.564639 | < 0.0001 | 6.000279 | 5.290208 | 6.805659 | < 0.0001 | 5.545907 | 4.897587 | 6.280049 | < 0.0001 |
| **icu_type_Medical_Surgical_Intensive_Care_Unit_MICU_SICU** | 3.421408 | 3.054652 | 3.832197 | < 0.0001 | 3.432689 | 3.065698 | 3.843614 | < 0.0001 | 3.256409 | 2.915319 | 3.637405 | < 0.0001 |
| **icu_type_Surgical_Intensive_Care_Unit_SICU** | 4.274925 | 3.827793 | 4.774289 | < 0.0001 | 4.329564 | 3.876183 | 4.835974 | < 0.0001 | 3.753547 | 3.365366 | 4.186504 | < 0.0001 |
| **icu_type_Neuro_Stepdown** | 5.691587 | 4.713845 | 6.87213 | < 0.0001 | 5.77973 | 4.798511 | 6.961592 | < 0.0001 | 6.256678 | 5.213469 | 7.508631 | < 0.0001 |
| **icu_type_Trauma_SICU_TSICU** | 3.022387 | 2.675229 | 3.414596 | < 0.0001 | 3.905111 | 3.472301 | 4.391869 | < 0.0001 | 3.172979 | 2.818181 | 3.572444 | < 0.0001 |
| **icu_type_other** | 8.718898 | 6.837431 | 11.118091 | < 0.0001 | 2.797011 | 1.448289 | 5.401733 | 0.0022 | 7.419958 | 3.407403 | 16.157691 | < 0.0001 |
| **mania_bipolar_diagnosis** | 1.209264 | 1.042456 | 1.402765 | 0.012 | 1.463521 | 1.237686 | 1.730563 | < 0.0001 | 1.283653 | 1.103899 | 1.492677 | 0.0012 |
| **icu_type_Neuro_Surgical_Intensive_Care_Unit_Neuro_SICU** | 4.451616 | 3.698196 | 5.358528 | < 0.0001 | 7.316587 | 6.107758 | 8.764664 | < 0.0001 | 7.714199 | 6.42806 | 9.257672 | < 0.0001 |
| **non_english_language** | 1.162431 | 1.053355 | 1.282803 | 0.0028 | 1.07034 | 0.987739 | 1.15985 | 0.1 | 1.248393 | 1.14853 | 1.356938 | < 0.0001 |
| **psychosis_diagnosis** | 1.340405 | 1.180066 | 1.52253 | < 0.0001 | 1.477116 | 1.268314 | 1.720292 | < 0.0001 | 0.953046 | 0.828869 | 1.095825 | 0.5 |
| **substance_use_diagnosis** | 1.530672 | 1.431952 | 1.636198 | < 0.0001 | 1.429826 | 1.336271 | 1.529931 | < 0.0001 | 1.541526 | 1.441655 | 1.648316 | < 0.0001 |
| **time_period_2014 - 2016** | 1.582632 | 1.482522 | 1.689501 | < 0.0001 | 1.553049 | 1.455273 | 1.657394 | < 0.0001 | 1.484982 | 1.392093 | 1.58407 | < 0.0001 |
| **time_period_2017 - 2019** | 1.341937 | 1.247327 | 1.443723 | < 0.0001 | 1.442131 | 1.343148 | 1.548409 | < 0.0001 | 1.253605 | 1.167231 | 1.34637 | < 0.0001 |
| **time_period_2020 - 2022** | 1.568321 | 1.446551 | 1.700342 | < 0.0001 | 1.289642 | 1.191105 | 1.396331 | < 0.0001 | 1.480721 | 1.36616 | 1.60489 | < 0.0001 |
| **ventilated_day1** | 2.222061 | 2.012853 | 2.453014 | < 0.0001 | 2.057382 | 1.867758 | 2.266256 | < 0.0001 | 1.992064 | 1.80698 | 2.196105 | < 0.0001 |

​​
